# Abscisic acid inhibits hypocotyl elongation acting on gibberellins, DELLA proteins and auxin

**DOI:** 10.1093/aobpla/ply061

**Published:** 2018-10-05

**Authors:** Riccardo Lorrai, Alessandra Boccaccini, Veronica Ruta, Marco Possenti, Paolo Costantino, Paola Vittorioso

**Affiliations:** 1 Department of Biology and Biotechnology, Sapienza University of Rome, Laboratory affiliated to Istituto Pasteur Italia – Fondazione Cenci Bolognetti, Rome, Italy; 2 Research Centre for Genomics and Bioinformatics, Council for Agricultural Research and Economics (CREA), Rome, Italy

**Keywords:** ABA, *Arabidopsis*, auxin, DELLA proteins, GA, hypocotyl elongation, PIF proteins

## Abstract

Hypocotyl elongation of *Arabidopsis* seedlings is influenced by light and numerous growth factors. Light induces inhibition of hypocotyl elongation (photomorphogenesis), whereas in the dark hypocotyl elongation is promoted (skotomorphogenesis). Abscisic acid (ABA) plays a major role in inhibition of hypocotyl elongation, but the molecular mechanism remains unclear. We investigated the effect of ABA during photo- and skotomorphogenesis, making use of appropriate mutants, and we show that ABA negatively controls hypocotyl elongation acting on gibberellin (GA) metabolic genes, increasing the amount of the DELLA proteins GAI and RGA, thus affecting GA signalling, and (ultimately) repressing auxin biosynthetic genes.

## Teaser

In this manuscript, we provide a molecular framework for the effect of abscisic acid (ABA) on hypocotyl elongation in *Arabidopsis* seedlings, independently of light conditions. Our results show that ABA negatively controls hypocotyl elongation by acting on gibberellin (GA) metabolic genes, increasing the amount of the DELLA proteins, thus affecting GA signalling, and (ultimately) repressing auxin biosynthetic genes.

## Introduction

Germination of seeds leads to the emergence of young seedlings, which undergo skotomorphogenic or photomorphogenic development depending on whether they grow in the dark or in the light, respectively. Dark-grown (etiolated) seedlings are characterized by long hypocotyls and small closed cotyledons; light-grown (de-etiolated) seedlings have short hypocotyls and open expanded cotyledons.

The phytochromes (phy), red and far-red light photoreceptors, in *Arabidopsis thaliana* are encoded by a small gene family (*phyA*–*phyE*), with phyB as the main switch from skotomorphogenic to photomorphogenic growth (Neff *et al.* 2000; Quail 2002). Once activated by red light, phyB translocates to the nucleus (Kircher *et al.* 2002) where it phosphorylates the PHYTOCHROME-INTERACTING FACTOR (PIF) proteins, which are subsequently degraded (Ni *et al.* 1999; Al-Sady *et al.* 2006). In the absence of light, PIF proteins accumulate in the nucleus and inhibit photomorphogenesis (Shin *et al.* 2009).

PHYTOCHROME-INTERACTING FACTOR 4 (PIF4) interacts with the AUXIN RESPONSE FACTOR (ARF) ARF6 and ARF8 to stimulate hypocotyl elongation (Oh *et al.* 2014). Auxin induces degradation of the ARF-inhibitor proteins AUX/IAA (Chapman and Estelle 2009; Vernoux *et al.* 2011), thus promoting hypocotyl elongation in the dark.

Gibberellins (GAs) repress photomorphogenesis, promoting hypocotyl elongation (Alabadí *et al.* 2004). Once GAs are bound to the GA INSENSITIVE DWARF1 (GID1) receptor, the GA–GID1 complex is able to interact with DELLA proteins and induce their degradation (Silverstone *et al.* 2001; Fu *et al.* 2002). The DELLA proteins are repressors of GA signalling, and they negatively control growth through the interaction with the PIF proteins (de Lucas *et al.* 2008; Feng *et al.* 2008) abolishing their transcriptional activity and promoting their degradation via the ubiquitin-proteasome system (Li *et al.* 2016).

Among plant growth processes, hypocotyl elongation has attracted much attention because of the simplicity of the organ, and because numerous plant growth factors affect this process, namely GAs and auxin as mentioned above, but also ethylene, brassinosteroids (Vandenbussche *et al.* 2005) and abscisic acid (ABA). As of this latter, an inhibiting effect on elongation in etiolated squash hypocotyl segments has been reported (Wakabayashi *et al.* 1989); more recently, it has been suggested that in etiolated *Arabidopsis* seedlings ABA suppresses hypocotyl elongation through the inhibition of an auxin-induced plasma membrane H^+^-ATPase (Hayashi *et al.* 2014). Coherently, it has been shown that ABA-responsive genes are repressed in shade avoidance-driven hypocotyl elongation (Kohnen *et al.* 2016).

Abscisic acid regulates several aspects of plant life including seed dormancy and germination, cell division and elongation, response to biotic and abiotic stresses, stomatal closure and fruit abscision. Given the multiplicity of its functions, ABA levels are checked through constant control of the ratio between synthesis and catabolism, in particular via the regulation of the genes encoding the nine-*cis*-epoxycarotenoid-dioxygenase 6 (NCED) and ABA-8C-hydroxylases (CYP707A) enzymes (Finkelstein 2013).

Abscisic acid is perceived through the PYRABACTIN RESISTANT (PYR), PYR-like (PYL) or REGULATORY COMPONENT of ABA RECEPTOR (RCAR) receptors. Once ABA is bound to these receptors, a complex is formed with the PROTEIN PHOSPHATASE 2Cs (PP2Cs), whose phosphatase activity is thus inhibited. In the absence of ABA, PP2Cs dephosphorylate SUCROSE NONFERMENTING 1 (SNF1)-RELATED PROTEIN KINASES (SnRK2s) which in turn activate downstream transcription factors and a number of ABA-responsive genes (Finkelstein 2013; Yang *et al.* 2017).

Abscisic acid is known as a growth-inhibiting hormone, although some papers described a promoting effect in maize, wheat, rice and *Arabidopsis* (Takahashi 1972; McWha and Jackson 1976; Barrero *et al.* 2008). In addition, it has been recently reported that the effect of ABA may be stimulatory or inhibitory depending on the dose and tissue sensitivity (Humplík *et al.* 2017). Thus, the function of ABA on hypocotyl elongation is still controversial, and so far it has not been elucidated the molecular mechanism underlying its action. Moreover, the interplay between ABA and endogenous (hormones) and/or environmental (light) cues involved in this process is still elusive.

Therefore, we investigated the effect of ABA on hypocotyl elongation during photo- and skotomorphogenesis to unravel whether the effect of ABA on hypocotyl growth is light-dependent or if ABA negatively controls cell expansion in hypocotyl both in skoto- and photomorphogenesis. Here, we show that in *Arabidopsis* seedlings ABA down-regulates GA biosynthetic genes and up-regulates catabolic ones, independently of light conditions. We also show that ABA stabilizes DELLA proteins in red light and that it down-regulates auxin biosynthetic genes. Finally, we show that ABA is not effective in a multiple *pif* mutant.

We propose a model of the action of ABA in controlling hypocotyl elongation that links all these evidences.

## Methods

### Plant material and growth conditions

All *Arabidopsis thaliana* lines used in this work were usually grown in a growth chamber at 22 °C with 16/8-h day/night cycles and light intensity of 300 μmol m^−2^ s^−1^ as previously described (Papi *et al.* 2000), unless otherwise noted. Seeds were surface sterilized and plated on MS agar (half-strength MS, 0.8 % agar, pH 5.7) and stratified at 4 °C for 3 days in the dark. The *aba1-5* and *aba2-1* mutants were kindly provided by Dr L. Lopez-Molina. *pif* quadruple mutant was obtained from the European Arabidopsis Stock Centre (NASC), whereas the *della* mutant was kindly provided by Prof. X. W. Deng. The corresponding wild-type lines were used (Columbia for *pif* quadruple mutant and Landsberg for the pentuple *della* mutant). The *DR5::GUS* line is the one described in Ulmasov *et al.* (1997).

### Phenotypic analysis

**Figure 1.**
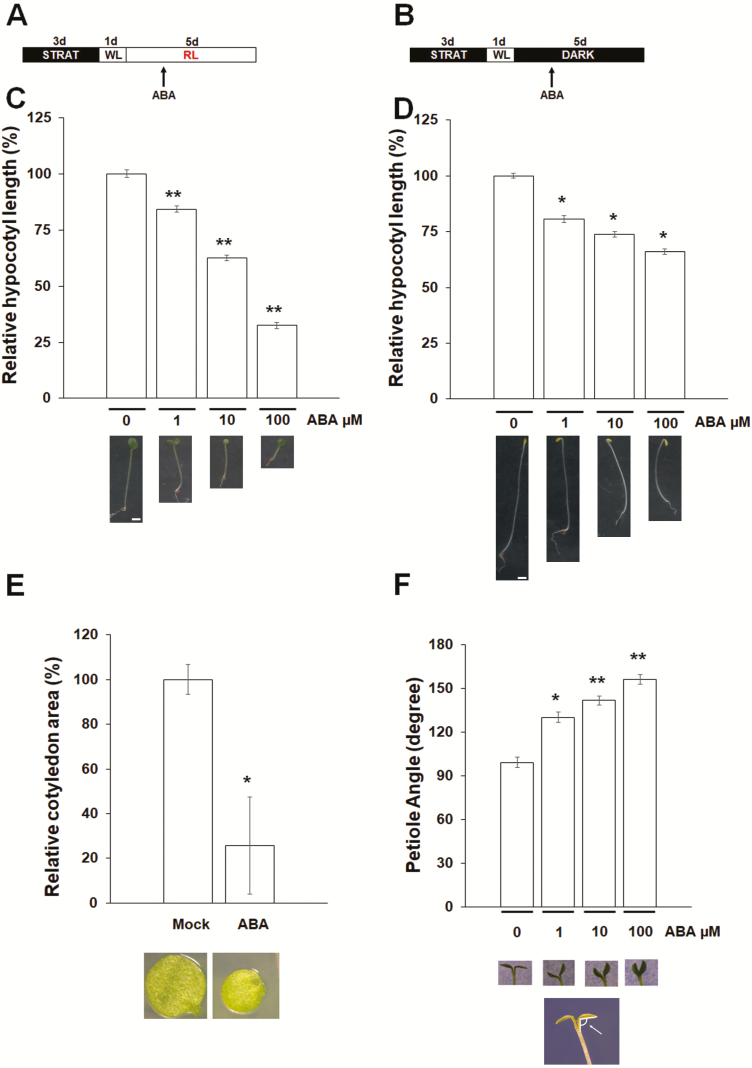
Abscisic acid (ABA) represses hypocotyl elongation and cotyledon expansion under red light (RL) and in the dark. (A and B) Diagram of ABA treatment, under RL (A), or in the dark (B). Wild-type (Col-0) seedlings were transferred on increasing ABA concentrations 60 h after induction of germination (arrow). WL, white light. (C and D) Relative hypocotyl length of 6-day-old seedlings grown under RL (C) or in the dark (D), in the presence of ABA. (E) Relative cotyledon area of mock- or ABA-treated (10 µM) 6-day-old seedlings. (F) Petiole angle of 6-day-old wild-type seedlings in the presence of increasing ABA concentrations. Top: histograms. Bottom: images of the seedlings in the different conditions. The scale bar indicates 1 mm. The measure of the angle between hypocotyl and petiole was performed as illustrated in the scheme. The values are the mean of three biological replicates, with SD values. Significant differences were analysed by *t*-test respect to the control (*P ≤ 0.05; **P ≤ 0.01).

For hypocotyl elongation, cotyledon expansion and bending analysis, the samples were first grown in white light (300 μmol m^−2^ s^−1^) (CCT 5700 K) for 24 h, then exposed to continuous monochromatic red light (660 nM) (40 μmol m^−2^ s^−1^) (mounting Heliospectra LX60 lamp) in a growth chamber at 22 °C. Hypocotyl length and cotyledon area were measured 6 days after stratification. Cotyledon area was measured at 10 μM ABA, because 1 μM ABA does not affect cotyledon expansion significantly, whereas 100 μM ABA results in cup-shaped cotyledons which make measurements not reliable. Cotyledon opening was analysed by measuring the angle between hypocotyl and petiole as reported in Fig. 1E.

For hypocotyl elongation in darkness the samples were first grown in white light for 24 h, then wrapped in several aluminium sheets for 4 days. For ABA and GA treatments, seeds were sown on MS agar with one layer of filter paper 595 (Schleicher & Schull, Dassel, Germany), then, 60 h after light exposure, seedlings were transferred to plates containing ABA (10 μM) (Duchefa A0941.0250) and different GA_4+7_ concentrations (1, 10, 100 μM) (Duchefa 0941) or mock (0.1 % ethanol). Hypocotyl length, cotyledon area and bending were measured using IMAGEJ software (Biorad). Both ABA and GA stock solutions were dissolved in ethanol, and the final ethanol concentration was 0.1 % for all the ABA and GA dilutions, as it was in mock.

The values are the mean of three biological replicates presented with SD values. Significant differences were analysed by *t*-test (*P ≤ 0.05; **P ≤ 0.01).

### GUS analysis

The analysis was performed on *DR5::GUS* stratified seedlings, exposed 24 h to white light, then grown 2 days under red light, and subsequently treated with ABA (100 μM) or mock (0.1 % ethanol), for 24 h under red light. Histochemical staining and microscopic analysis were carried out according to Capone *et al.* (1991), except that seedlings were incubated at 37 °C for 12 h. Stained seedlings (after washing in 70 % ethanol) were analysed and photographed under an Axioskop 2 plus microscope (Zeiss).

### Transcript analysis

Wild-type (Col-0) seedlings were grown under white light for 3 days before ABA treatment, then seedlings were moved in monochromatic red light or in the dark, with ABA (100 μM), or mock (0.1 % ethanol), for 4 h in liquid medium.

We have analysed gene expression and protein level 4 days after treatment, because we figured that the molecular mechanism would precede the phenotypic effect detectable after 6 days of treatment. We used 100 μM ABA because with this ABA concentration we observed the strongest phenotype.

Total RNA was extracted and purified according to Vittorioso *et al.* (1998). RT–qPCR assays were performed according to Gabriele *et al.* (2010). Relative expression levels were normalized with *UBQ10* (At4g05320) reference gene, and are presented by the ratio of the corresponding mRNA level of the mock-treated sample, which was set to 1. The primers used are listed in Table 1. The values of relative expression levels are the mean of three biological replicates presented with SD values. Significant differences were analysed by *t*-test (*P ≤ 0.05; **P ≤ 0.01).

**Table 1.** List of the primers used in gene expression analysis.

Gene name	Forward	Reverse
*AtGA3ox1*	GCTTAAGTCTGCTCGGTCGG	AGTGCGATACGAGCGACG
*AtGA3ox2*	ACGTCGGTGACTTGCTCCA	GTTAACCCTGGCTCGGTGAA
*AtGA2ox2*	TCCGACCCGAACTCATGACT	CGGCCCGGTTTTTAAGAGAC
*AtGA2ox4*	CTCTTTCGGCGATGGTTATG	AAACGGCTATCCTCAAGTCG
*KAO1*	TCGACCCTGAAGTCTTTCCA	TCGACCCTGAAGTCTTTCCA
*AtGA20ox1*	AGCGAGAGGAAATCACTTGC	AGCGAGAGGAAATCACTTGC
*AtGA20ox2*	TGCCAAACACCAGATCTCAC	TGCCAAACACCAGATCTCAC
*UBQ10*	GGCCTTGTATAATCCTGATGAATAAG	GGCCTTGTATAATCCTGATGAATAAG
*YUC3*	GAAGGCAGCGACATTTTCTC	TACCCCTTCACGTTTCAAGC
*YUC5*	GGGTTAACGGTCCTGTAATCGT	TCTGCTCTCTCCAATACCACAAAG
*YUC6*	AGGTCCACTCGAGCTCAAAA	CCTTCTTATCCCCGAACACA

### Immunoblot analysis

Total proteins were extracted from seedlings treated with ABA (100 μM) or ethanol (mock treatment) according to Gabriele *et al.* (2010). Seedlings were grown in white light condition for 3 days before ABA treatment, to allow GAI and RGA accumulation. Then seedlings were moved in monochromatic red light with ABA (100 μM), or mock (0.1 % ethanol), for 4 h in liquid medium. A total of 30–40 μg of protein extract was separated on SDS–polyacrylamide gel and blotted on a PVDF Immobilon-P transfer membrane (Millipore). Detection of proteins was performed with anti-GAI or anti-RGA antibodies (Agrisera, Vännäs, Sweden) as primary antibody and peroxidase-conjugated anti-rabbit as secondary antibody (Sigma, St. Louis, MO, USA). H3 was detected using an anti-H3 antibody (Biorbyt, Cambridge, UK). For densitometric analysis, mean intensities were background subtracted and normalized to the loading control using the ImageLab software v. 5.2.1. The values are the mean of three biological replicates presented with SD values. Significant differences were analysed by *t*-test (*P ≤ 0.05; **P ≤ 0.01).

### Statistical analysis

Each experiment was performed in triplicate and repeated with three biological replicates. Results are expressed as mean ± SD. Two-tailed Student’s *t*-test was used to evaluate statistical significance (*P ≤ 0.05; **P ≤ 0.01).

## Results and Discussion

### ABA represses hypocotyl elongation and cotyledon expansion independently of light conditions

To investigate the effect of ABA on hypocotyl elongation during photo- or skotomorphogenesis, we analysed the response of hypocotyl elongation to ABA treatment on seedlings grown under red light or in the dark (Fig. 1A and B). When treated with increasing concentrations of ABA, wild-type (Col-0) seedlings grown under red light or in the dark were significantly shorter than controls in a dose-dependent manner (Fig. 1C and D). In addition, we compared the hypocotyl length of the ABA biosynthetic mutants *aba1-5* and *aba2-1*, which are affected in ABA content (Leon-Kloosterziel *et al.* 1996). We measured hypocotyl length of *aba1-5* and *aba2-1* seedlings grown under red light or in the dark. This analysis showed that the hypocotyls of both mutants were not significantly longer than the wild type **[see****Supporting** Information—**Fig. S1****]**, probably due to the residual ABA content. It should also be noted that these mutants have been previously shown to display reduced growth, because of their inability to retain water, as a result of impaired stomatal closure (Cheng *et al.* 2002; Barrero *et al.* 2005).

We then examined whether ABA would inhibit also cotyledon expansion. Cotyledon area of ABA-treated seedlings was significantly reduced compared to mock-treated controls (Fig. 1E). Cotyledon opening was also inhibited by ABA treatment: the angle between hypocotyl and petiole significantly increased in ABA-treated seedlings in a dose-dependent manner (Fig. 1F).

### ABA represses GA metabolism and stabilizes DELLA proteins

**Figure 2.**
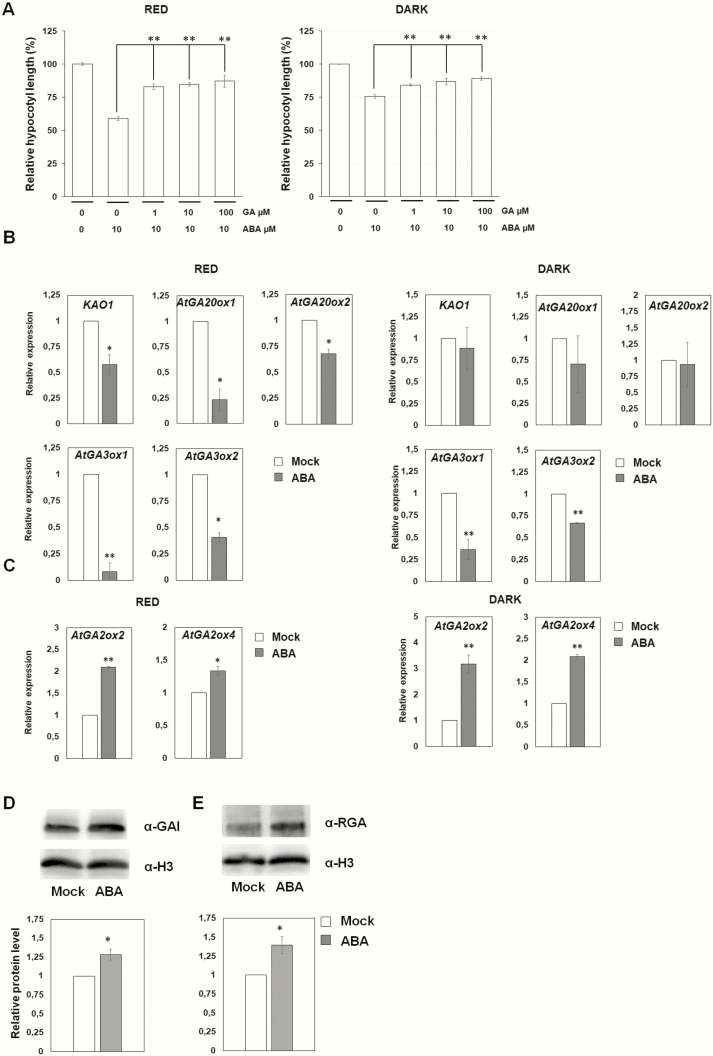
Abscisic acid (ABA) acts through GAs independently of light conditions. (A) Relative hypocotyl length of 6-day-old wild-type seedlings (Col-0) treated with ABA plus GA under red light and in the dark. (B and C) Relative expression level of the GAs biosynthetic (B) and catabolic (C) genes, under red light (left), or in the dark (right). (D and E) Protein level of GAI (D) and RGA (E). Proteins are from 4-day-old wild-type (Col-0) seedlings, grown under red light, mock- or ABA-treated (4 h; 100 µM). H3 used as loading control. Western blot (top) and densitometric analysis (bottom).

As GAs promote hypocotyl elongation (Alabadí *et al.* 2004), we verified whether they might counteract the effect of ABA; measurement of hypocotyl length of wild-type (Col-0) seedlings treated with ABA and increasing amounts of GAs revealed that 10 µM GA was sufficient to revert, though not completely, the effect of ABA (Fig. 2A).

To assess whether ABA inhibits hypocotyl elongation acting on GA metabolism, we measured by RT–qPCR the transcription of a number of GA metabolic genes on 4-day-old wild-type (Col-0) seedlings treated for 4 h with ABA, under red light or in the dark, compared to mock-treated controls. The genes analysed were the GA biosynthetic genes encoding *ent*-kaurenoic acid oxidase (*KAO1*), GA20-oxidase1 and GA20-oxidase2 (*AtGA20ox1* and *2*), GA3-oxidase1 and GA3-oxidase2 (*AtGA3ox1* and *2*); and the GA catabolic genes encoding GA2-oxidase2 and GA2-oxidase4 (*AtGA2ox2* and *4*).

In red light conditions, all the GA biosynthetic genes were significantly down-regulated by ABA treatment: the expression levels of *AtGA3ox1*, *AtGA3ox2*, *AtGA20ox1*, *AtGA20ox2* and *KAO1* were, respectively, 12.5-, 2.5-, 4.3-, 1.5- and 1.7-fold lower than mock-treated controls; in the dark, the expression levels of *AtGA3ox1* and *AtGA3ox2* were, respectively, 2.7- and 1.5-fold lower than mock-treated controls, whereas *AtGA20ox1*, *AtGA20ox2* and *KAO1* were not significantly different from the mock-treated control (Fig. 2B). The catabolic genes *AtGA2ox2* and *AtGA2ox4* showed an increased expression level, 2.1- and, respectively, 3.1-fold over mock-treated controls in red light; and 1.3- and, respectively, 2.1-fold in the dark (Fig. 2C). Although, among the biosynthetic genes, only *AtGA3ox1* and *AtGA3ox2* were repressed by ABA in the dark, it should emphasized that these are the key genes for the control of GA biosynthesis (Mitchum *et al.* 2006). Therefore, these results suggest that ABA inhibits hypocotyl elongation acting on GA metabolism, independently of light conditions.

The DELLA proteins GA INSENSITIVE (GAI) and REPRESSOR OF *ga1-3* (RGA), GA-signalling repressors, inhibit hypocotyl elongation under red light (de Lucas *et al.* 2008; Feng *et al.* 2008). Gibberellins trigger proteasome-mediated degradation of these DELLA proteins, thus relieving their inhibitory effect on hypocotyl elongation, under white light (King *et al.* 2001). Since we observed that ABA affects GA metabolic genes, we verified whether ABA treatment leads to the stabilization of GAI and RGA. Immunoblot analysis of ABA-treated wild-type (Col-0) seedlings (4 h treatment) revealed that, under red light, addition of ABA significantly increased the level of both GAI and RGA proteins, compared to mock-treated seedlings (Fig. 2D and E), suggesting that ABA inhibits hypocotyl elongation by reducing the level of GAs and consequently increasing the level of GAI and RGA. In the dark, the level of both GAI and RGA proteins was undetectable, both in ABA-treated and mock-treated seedlings **[see****Supporting Information—Fig. S2****]**, probably due to high GA level, as already reported (Alabadì *et al*. 2008).

**Figure 3.**
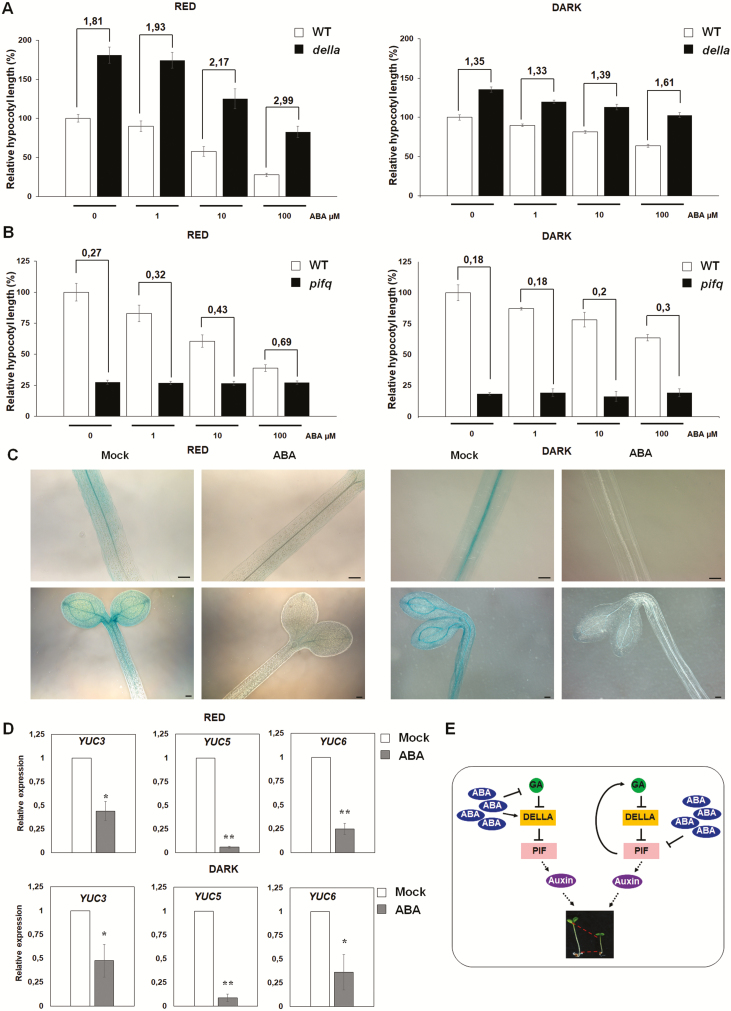
Abscisic acid (ABA) lowers auxin level through PIF proteins. (A and B) Relative hypocotyl length of 6-day-old pentuple *della* mutant (A) and quadruple *pif* mutant (B) respect to the wild type (Col-0), under red light (left), or in the dark (right). The ratio of the hypocotyl length of the mutants to wild-type seedlings (Col-0) is shown. Abscisic acid treatment as reported in Fig. 1A. (C) Histochemical staining of 3-day-old *DR5::GUS* mock- or ABA-treated (100 µM) seedlings, grown under red light (left) or in the dark (right). The horizontal bar corresponds to 100 μm. (D) Relative expression level of the auxin biosynthetic genes under red light (top), or in the dark (bottom). Relative expression levels were normalized with *UBQ10* (At4g05320) reference gene, and are presented by the ratio of the corresponding mRNA level of the mock-treated sample, which was set to 1. RNA are from 4-day-old wild-type (Col-0) seedlings, grown under red light or in the dark, mock- or ABA-treated (4 h; 100 µM). Values are the mean of three biological replicates, with SD. Significant differences analysed by *t*-test (*P ≤ 0.05; **P ≤ 0.01). (E) Schematic model of ABA function in the inhibition of hypocotyl elongation in a DELLA-dependent or -independent pathway; ABA represses GA biosynthesis, thus DELLA proteins are stabilized, and inhibit the activity of PIFs, ultimately repressing auxin biosynthesis (left). Alternatively, ABA inhibits PIF proteins, which no longer induce both GA and auxin biosynthesis (right).

We then wondered whether the hypocotyl response to ABA of the quintuple *della* mutant that lacks all five DELLA proteins and shows an increased hypocotyl length (Feng *et al.* 2008) would be impaired. Indeed, hypocotyls of ABA-treated quintuple *della* seedlings—under red light or in the dark—were less sensitive to ABA inhibition than hypocotyls of both mock-treated *della* mutants and ABA-treated wild type (Fig. 3A). However, the residual response to ABA of the quintuple *della* mutant suggests that ABA might act both via a DELLA-dependent and a DELLA-independent pathway.

### ABA acts through PIF proteins

In the dark, GAs repress photomorphogenesis (Alabadí *et al.* 2004). DELLA proteins are degraded in the presence of GAs; conversely, in the light the amount of DELLA proteins increases, as a consequence of reduced GAs (Achard *et al.* 2007). The molecular network underlying the switch between skoto- and photomorphogenesis also includes the PHYTOCHROME-INTERACTING FACTORS 1, 3, 4 and 5 (PIF1, PIF3, PIF4 and PIF5) (Oh *et al.* 2004; de Lucas *et al.* 2008; Feng *et al.* 2008, Leivar *et al.* 2008). DELLA proteins directly repress the transcriptional activity of PIF proteins (de Lucas *et al.* 2008; Feng *et al.* 2008), which in turn repress photomorphogenesis, as the *pif1pif3pif4pif5* mutant (*pifq*) shows a constitutive photomorphogenic phenotype (Leivar *et al.* 2008). Consistently, hypocotyl elongation of *pifq* mutant seedlings, grown under red light or in the dark, was insensitive to ABA, as hypocotyl length did not show differences between mock- and ABA-treated seedlings (Fig. 3B), thus indicating that ABA exerts its function via the repression of PIF activity through DELLA proteins.

PHYTOCHROME-INTERACTING FACTOR proteins promote hypocotyl elongation by directly inducing the expression of the auxin biosynthetic *YUCCA* (*YUC*) genes (Hornitschek *et al.* 2012). It has also been shown that the AUXIN RESPONSE FACTORS (ARFs) ARF6 and ARF8 cooperate with PIF4, through direct interaction, in positively controlling hypocotyl elongation (Oh *et al.* 2014). PHYTOCHROME-INTERACTING FACTOR 4 also induces the expression of auxin biosynthetic genes *YUC8*, *TAA1* and *CYB79B2* (Hornitschek *et al.* 2012), thus increasing auxin levels.

To assess whether ABA inhibits hypocotyl elongation acting on auxin, we utilized the synthetic auxin-response reporter *DR5::GUS*. As shown in Fig. 3C, GUS activity is reduced in ABA-treated *DR5::GUS* seedlings, grown under red light or in the dark. Consistently, the *YUC3*, *YUC5* and *YUC6* genes were significantly down-regulated by ABA, independently of light conditions (Fig. 3D), thus suggesting that ABA ultimately acts on auxin.

In conclusion, we show here that ABA represses GA biosynthetic genes while inducing GA catabolic ones, suggesting that ABA is likely to reduce GA levels thus stabilizing the DELLA proteins GAI and RGA. In addition, our results suggest that ABA exerts its effect via the repression of PIF (activity), as supported by the significantly decreased ABA sensitivity of the *pif* multiple mutant. Finally, we show that auxin biosynthetic genes are down-regulated by ABA treatment.

It is tempting to speculate on a model whereby ABA inhibits hypocotyl elongation by negatively regulating GAs, thus causing an increased level of DELLA proteins; this, possibly via a decreased activity of PIF proteins, results in a down-regulation of auxin biosynthetic genes and therefore inhibition of cell expansion. However, we cannot rule out the possibility that ABA may function in a DELLA-independent pathway, through inhibition of PIF proteins. Indeed, PIF proteins have been shown to induce GA biosynthetic genes (*GA20ox1* and *GA20ox2*) (Filo *et al.* 2015), thus destabilizing DELLA proteins (Fig. 3E).

Although we are far from having established a firm causal relation between the effects we report, we believe we have opened the way to much further interesting work to verify this novel pathway that would allow ABA to control cell expansion by ultimately acting on auxin.

## Conflict of Interest

None declared.

## Supporting Information

The following additional information is available in the online version of this article—

Figure S1. Hypocotyl length of *aba* mutants. Relative hypocotyl length of 6-day-old wild-type (Col-0), *aba1-5* and *aba2-1* seedlings under red light (A) and in the dark (B).

Figure S2. Protein level of GAI and RGA. Proteins are from 4-day-old dark-grown wild-type (Col-0) seedlings, mock- or ABA-treated (4 h; 100 µM). The *sleepy1* (*sly1*) mutant has been used as positive control, since it overacumulates DELLA proteins. The loss-of-function *gai-t6* mutant has been used as negative control. H3 used as loading control.

Supplementary_Infomation

## Sources of Funding

This research was supported by research grants from Ministero dell’Istruzione, Università e Ricerca, Progetti di Ricerca di Interesse Nazionale, and from Sapienza Università di Roma to P.C. and to P.V. and from Istituto Pasteur Italia-Fondazione Cenci Bolognetti to P.V.

## Contributions by the Authors

P.V. and R.L. conceived the strategy. P.V. directed the project. R.L. and A.B. designed the experiments. R.L., A.B., V.R. and M.P. performed the experiments. P.V. and P.C. wrote the manuscript. R.L., A.B. and V.R. revised the manuscript. All Authors read and approved the final manuscript.
