# Supplementary material for: Abscisic acid inhibits hypocotyl elongation acting on gibberellins, DELLA proteins and auxin
Source: AoB Plants. 2018 Oct 5;10(5):ply061. doi: 10.1093/aobpla/ply061 (PMC6204436; doi:10.1093/aobpla/ply061)
Supplement: Supplementary_Infomation [file ply061_suppl_supplementary_infomation.pdf]

Supporting Information Figure S1

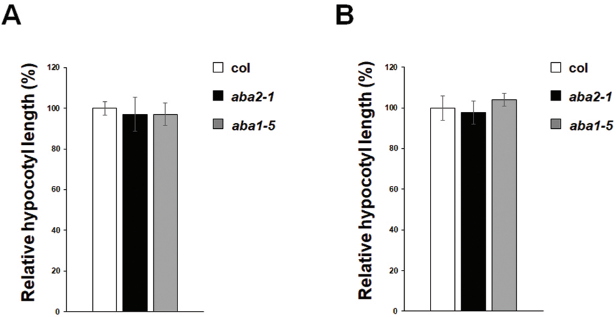

Supporting Figure S1. Hypocotyl length of *aba* mutants. Relative hypocotyl length of six days-old wild type (Col-0), *aba1-5* and *aba2-1* seedlings under red light (**A**) and in the dark (**B**).

Supporting Information Figure S2

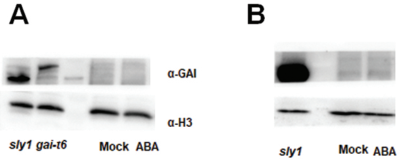

Supporting Figure S2. Protein level of GAI and RGA. Proteins are from four days-old dark-grown wild type (Col-0) seedlings, mock- or ABA-treated (4 h; 100 μM). The *sleepy1* (*sly1*) mutant has been used as positive control, since it overacumulates DELLA proteins. The loss of function *gai-t6* mutant has been used as negative control. H3 used as loading control.
